# Association between the European GWAS-Identified Susceptibility Locus at Chromosome 4p16 and the Risk of Atrial Septal Defect: A Case-Control Study in Southwest China and a Meta-Analysis

**DOI:** 10.1371/journal.pone.0123959

**Published:** 2015-04-13

**Authors:** Li Zhao, Bei Li, Ke Dian, Binwu Ying, Xiaojun Lu, Xuejiao Hu, Qi An, Chunxia Chen, Chunyan Huang, Bin Tan, Li Qin

**Affiliations:** 1 Department of Laboratory Medicine, West China Hospital, Sichuan University, Chengdu, P. R. China; 2 Department of Cardiothoracic Surgery, West China Hospital, Sichuan University, Chengdu, P.R. China; University of Illinois at Chicago, UNITED STATES

## Abstract

Atrial septal defect (ASD) is the third most frequent type of congenital heart anomaly, featuring shunting of blood between the two atria. Gene-environment interaction remains to be an acknowledged cause for ASD occurrence. A recent European genome-wide association study (GWAS) of congenital heart disease (CHD) identified 3 susceptibility SNPs at chromosome 4p16 associated with ASD: *rs870142*, *rs16835979* and *rs6824295*. A Chinese-GWAS of CHD conducted in the corresponding period did not reveal the 3 susceptibility SNPs, but reported 2 different risk SNPs: *rs2474937* and *rs1531070*. Therefore, we aimed to investigate the associations between the 3 European GWAS-identified susceptibility SNPs and ASD risk in the Han population in southwest China. Additionally, to increase the robustness of our current analysis, we conducted a meta-analysis combining published studies and our current case-control study. We performed association, linkage disequilibrium, and haplotype analysis among the 3 SNPs in 190 ASD cases and 225 age-, sex-, and ethnicity-matched healthy controls. Genotype and allele frequencies among the 3 SNPs showed statistically significant differences between the cases and controls. Our study found that individuals carrying the allele T of *rs870142*, the allele A of *rs16835979*, and the allele T of *rs6824295* had a respective 50.1% (odds ratio (OR) = 1.501, 95% confidence interval (CI) = 1.122-2.009, *P_FDR-BH_* = 0.018), 48.5% (OR = 1.485, 95%CI = 1.109-1.987, *P_FDR-BH_* = 0.012), and 38.6% (OR = 1.386, 95%CI = 1.042-1.844, *P_FDR-BH_* = 0.025) increased risk to develop ASD than wild-type allele carriers in our study cohort. In the haplotype analysis, we identified a disease-risk haplotype (TAT) (OR = 1.540, 95%CI = 1.030-2.380, *P_FDR-BH_* = 0.016). Our meta-analysis also showed that the investigated SNP was associated with ASD risk (combined OR (95%CI) = 1.35 (1.24-1.46), *P <* 0.00001). Our study provides compelling evidence to motivate better understanding of the etiology of ASD.

## Introduction

Congenital heart disease (CHD), characterized by cardiovascular structure and function abnormalities, is one of the most frequently occurring congenital malformations in infants and children. The incidence of CHD is ~8 per 1000 live births globally, and it is much higher in East Asia [1–4]. Atrial septal defect (ASD), the third most common type of CHD, is mainly caused by the hypoplasia of atrial septum, resulting in abnormal flow of blood between the systemic and pulmonary circulations. Despite this defect, ASD patients lack specific symptoms in the early stages so that diagnosis can be difficult. Thus, diagnosis based on pathogenic mechanisms is of particular importance.

The etiology of ASD is complex, involving genetic and environmental factors [5]. So far, numerous genes encoding transcription factors and important heart proteins have been associated with ASD risk. These include *GATA4*, a transcription factor essential for heart formation, and *TBX5*, a T-box protein required for cardiac conduction system, which have been reported as conferring predisposition to ASD occurrence [6,7]. In addition, mutations of *NKX2-5* and *NOTCH1* also have been associated with ASD risk [8]. Nonetheless, currently identified genetic factors only account for a small part of the etiology of ASD. More genes that are known to play a role in normal heart function need to be investigated for mutations that may be associated with alterations in heart development.

Genome-wide association study (GWAS) has emerged as an important method to reveal susceptibility genes of complex diseases and promoted medical progress. A recent European-GWAS of CHD (Cordell’s GWAS) did not uncover the susceptibility genes associated with all CHD phenotypes. However, when the 340 patients with ASD were analyzed separately, 3 SNPs at chromosome 4p16, *rs870142* (OR = 1.519, *P* = 9.52×10^-7^), *rs16835979* (OR = 1.511, *P* = 1.24×10^-6^), and *rs6824295* (OR = 1.505, *P* = 1.66×10^-6^), were found to influence the risk of ASD [9]. Interestingly, a Chinese-GWAS of CHD performed in the same period did not identify the 3 risk SNPs, but reported 2 different susceptibility SNPs associated with all CHD phenotypes (*rs2474937* and *rs1531070*)[10]. Whether the identified susceptibility locus in the European-GWAS of CHD contributes to ASD occurrence in the Han population in southwest China has not been elucidated. Therefore, we aimed to investigate the relationships between the European GWAS-identified susceptibility SNPs (*rs870142*, *rs16835979* and *rs6824295*) and ASD risk in a Han population in southwest China.

## Materials and Methods

### Study Subjects

We recruited a total of 190 unrelated individuals with ASD (median age 20 years; 69 males and 121 females) from the inpatient unit of the department of cardiac surgery in the West China Hospital of Sichuan University from September 2012 to February 2014. ASD cases were diagnosed by cardiovascular specialists on the basis of transthoracic or transesophageal echocardiographic examination and cardiac catheterization, and all of the cases were further confirmed by surgery. The controls (n = 225, 85 males and 140 females; median age 22 years) were sex-, age-, and ethnicity-matched healthy unrelated individuals selected from those coming to our hospital for regular health examinations, excluding those with abnormal physical examination results and any types of CHD.

All of the cases and controls were genetically unrelated Han-Chinese individuals living in Sichuan province of southwest China. At recruitment, each participant donated approximate 3 ml of blood for genomic DNA extraction.

All the study protocols were approved by the Ethics Committee of the West China Hospital of Sichuan University. The study conformed to the principles outlined in the declaration of Helsinki. All the subjects provided written informed consent prior to the beginning of the study.

### SNP Genotyping

Genomic DNA was extracted using a QIAamp DNA Blood mini kit (Qiagen, Germany) and diluted to10ng/μl with AE buffer, according to the manufacturer's instructions. The target fragments containing reference alleles from all of the study subjects were amplified by PCR with *rs870142* specific primers (5’-AGGACTG GGAAATTTGGGAAG-3’ (Forward); 5’-ACTTTCCCCTAAGAGTCCAGT-3’ (Reversed)), *rs16835979* specific primers (5’-AGTGAGAGTGTGGACTCTAGA ATGG-3’ (Forward); 5’- AATGAATGACACATGTGCAGC-3’ (Reversed)), and *rs6824295* specific primers (5’-CAGCCCTCCAGAGCAGCT-3’ (Forward); 5’- GGAGCGAGCAGACACAGT-3’ (Reversed)), respectively. The specific PCR amplifications and corresponding genotyping of the 3 SNPs were performed by the method of high-resolution melting (HRM) in the LightCycler 480 (Roche Diagnostics). Specific experimental procedures were performed using methods previously described in the literatures [11,12].

### Statistical analysis

Hardy—Weinberg equilibrium was evaluated for each group. Allele and genotype case/control association analysis was conducted using all the genotype data. For each SNP, we calculated empirical significance values on the basis of 10,000 permutations. This ensures that deviation from small sample size will not cause false positives.

To assess whether haplotype further increased ASD risk, compared with single-SNP analysis, we performed linkage disequilibrium and haplotype association analysis among the 3 SNPs. All the statistical analysis was performed by the software PLINK version 1.07 (http://pngu.mgh.harvard.edu/~purcell/plink). Additionally, a “conditional” test was performed for each SNP in PLINK, to evaluate whether the haplotype associations could be attributed to a single SNP (i.e., testing the haplotype effect after conditioning on the effect of each single SNP).

In this study, we utilized false discovery rate (FDR-BH) method to correct the *P* value when multiple comparisons existed. *P* values were two sides and corrected *P (P*_*FDR-BH*_ *)* <0.05 was considered to be statistically significant.

### Meta-analysis

We also conducted a meta-analysis combining published studies and our current case-control study for further evaluation of the associations between the 3 SNPs and the risk of ASD. We searched MEDLINE, EMBASE, Cochrane library, and Chinese databases (CNKI, CQVIP and Wan-fang Databases) to collect the related literatures published in English and Chinese between January 2007 and January 2015, utilizing the theme words “congenital heart disease” “atrial septal defect”, “ASD”, “*rs870142*”, “*rs16835979*”, “*rs6824295*”, and “genetic polymorphism”. The inclusion criteria were: (1) Studies evaluating the association between *rs870142*, *rs16835979*, *rs6824295* polymorphisms and ASD risk; (2) Available data for calculating allelic odds ratio (ORs) with corresponding 95% confidence interval (95% CI); (3) Genotypes in controls conforming to Hardy-Weinberg equilibrium (*P*>0.05). Reviews and case reports were excluded.

The following data were extracted from each eligible study: first author's name, year of publication, study design, geographic location or ethnicity of study population, sample size, frequencies of allele in cases and controls. Heterogeneity across all eligible studies was estimated by the Cochran's *Q* statistic. Heterogeneity was considered evident at *P*<0.05 for the Q statistic. Random-effect model was used when the heterogeneity among studies existed; otherwise, fixed-model was utilized. The allele model with combined ORs with 95% CIs was used to assess the associations between the investigated 3 SNPs and ASD risk. *P* values were two sides and *P* <0.05 was considered to be statistically significant. The meta-analysis was performed by the software Review Manager Version 5.3 (http://www.cc-ims.net/RevMan).

## Results

### Associations of *rs870142* (T > C), *rs16835979* (A > C) and *rs6824295* (T > C) polymorphisms with ASD risk

The genotypic distribution did not deviate from the Hardy-Weinberg equilibrium for the 3 susceptibility SNPs (*rs870142*, *rs16835979* and *rs6824295*) in the cases and controls. There were statistically significant differences in genotype and allele frequencies between the ASD cases and controls for each of the 3 variants (*rs870142*: χ^2^ = 10.52, *P*_*FDR-BH*_ = 0.015 and χ^2^ = 7.52, *P*_*FDR-BH*_ = 0.018; *rs16835979*: χ^2^ = 10.09, *P*_*FDR-BH*_ = 0.009 and χ^2^ = 7.09, *P*_*FDR-BH*_ = 0.012; *rs6824295*: χ^2^ = 8.62, *P*_*FDR-BH*_ = 0.013 and χ^2^ = 5.03, *P*_*FDR-BH*_ = 0.025, respectively).

The T-allele and TT-genotype of *rs870142* variant were more frequent in the cases than in the controls (37.6% vs. 28.7% and 15.8% vs. 6.2%). Individuals carrying the *rs870142* T-allele showed a 50.1% (OR = 1.501, 95%CI = 1.122–2.009, *P*_*FDR-BH*_ = 0.018) increased ASD risk. Those with the TT-genotype had an increased risk of ASD with an OR of 2.826 (95%CI = 1.451–5.505, *P*_*FDR-BH*_ = 0.006) using a recessive genetic model. Similarly, for the *rs16835979* polymorphism, we observed that the frequencies of the A-allele and AA-genotype were higher in the cases than in the controls, with the OR of an A allele carrier equaling 1.485 (95%CI = 1.109–1.987, *P*_*FDR-BH*_ = 0.012) and the OR of an AA genotype carrier equaling 2.730 (95%CI = 1.425–5.228, *P*_*FDR-BH*_ = 0.003). In addition, the frequencies of the T-allele and TT-genotype of the *rs6824295* polymorphism were overrepresented in the cases compared with the controls. The *rs6824295* (T > C) mutation conferred an OR (95%CI) of 1.386 (1.042–1.844) per copy of the T allele in the additive genetic model and an OR (95%CI) of 2.478 (1.328–4.623) per copy of the TT genotype using a recessive genetic model. The specific results are summarized in Table 1.

**Table 1:** Association of *rs870142* (T>C), *rs16835979* (A>C) and *rs6824295* (T>C) polymorphisms with ASD.

SNP	Genotype frequency n (%)			Allele frequency n (%)		OR(95%CI)	*P*	*P*_*perm*_	*P*_*FDR-BH*_
*rs870142*	CC	TC	TT	T	C				
Cases(190)	77(40.5)	83(43.7)	30(15.8)	143(37.6)	237(62.4)	2.826(1.451–5.505)^*a*^	0.002	0.003	0.006
Controls(225)	110(48.9)	101(44.9)	14(6.2)	129(28.7)	321(71.3)	1.501(1.122–2.009)^b^	0.006	0.006	0.018
*rs16835979*	CC	AC	AA	A	C				
Cases (190)	79(41.6)	80(42.1)	31(16.3)	142(37.4)	238(62.6)	2.730(1.425–5.228)^*a*^	0.002	0.003	0.003
Controls(225)	111(49.3)	99(44.0)	15(6.7)	129(28.7)	321(71.3)	1.485(1.109–1.987)^b^	0.008	0.009	0.012
*rs6824295*	CC	TC	TT	T	C				
Cases (190)	72(40.7)	86(42.2)	32(17.1)	150(39.5)	230(60.5)	2.478(1.328–4.623)^*a*^	0.003	0.004	0.003
Controls (225)	98(50.0)	110(42.0)	17(8.0)	144(32.0)	306(68.0)	1.386(1.042–1.844)^b^	0.025	0.028	0.025

^a^ the value of OR (95%CI) in the recessive genetic model.

^b^ the value of OR (95%CI) in the additive genetic model. P_perm_ denoted empirical significance values on the basis of 10,000 permutations in the recessive and additive genetic models, respectively. P_FDR-BH_ denoted the P-value after performing multiple testing corrections with FDR-BH method.

### Haplotype analysis among the 3 SNPs at chromosome 4p16

As shown in Table 2, the 3 SNPs of *rs870142*, *rs16835979* and *rs6824295* were in linkage disequilibrium with one another. Haplotype analysis showed that haplotype TAT (carried by 36.2% of the ASD cases, versus 26.7% of the controls) was associated with the ASD risk (OR = 1.54, 95%CI = 1.030–2.380, *P*_*FDR-BH*_ = 0.016). In the conditional haplotype-based analysis, we found that the haplotype association could all be accounted for by any one of the SNPs (*rs870142*: χ^2^ = 0.33, *P* = 0.85; *rs16835979*: χ^2^ = 1.13, *P* = 0.57; *rs6824295*: χ^2^ = 2.78, *P* = 0.25). From the significance level, we may conclude that the haplotype association could be most likely accounted for by the *rs870142*. The specific results are shown in Table 3.

**Table 2:** Results of linkage disequilibrium analysis among the 3 SNPs at chromosome4p16.

r2	*rs16835979*	*rs6824295*
*rs870142*	0.845	0.736
*rs16835979*		0.722

**Table 3:** Haplotype main effect and conditional haplotype-based analysis tests (MHF>0.01).

Haplotype	S1	S2	S3	F(case/control)	χ^2^	OR (95%CI)	Asymptotic *P*-value	*P*_*FDR-BH*_
H1	T	A	T	0.362/0.267	8.424	1.540 (1.030–2.380)	0.004	0.016
H2	C	C	T	0.031/0.047	1.386		0.239	0.319
H3	C	A	C	0.010/0.012	0.260		0.610	0.610
H4	C	C	C	0.599/0.674	4.892	0.720 (0.490–0.910)	0.027	0.054
P_cond_	0.85	0.57	0.25					

MHF: minor haplotype frequency; H1, H2, H3, H4 represented haplotype1, haplotype2, haplotype3, haplotype4, respectively. S1, S2, S3 represented SNP rs870142, rs16835979, rs6824295, respectively. F(case/control) represented haplotype frequency in the cases and controls. OR (95%CI) and asymptotic P-value denoted haplotypic odds ratio and P-value in the haplotype association analysis. P_FDR-BH_ denoted the P-value after performing multiple testing corrections with FDR-BH method. P_cond_ denoted P-value in the conditional test which determined whether the haplotype association could be attributive to that single SNP.

### The results of Meta-analysis

**Fig 1:**
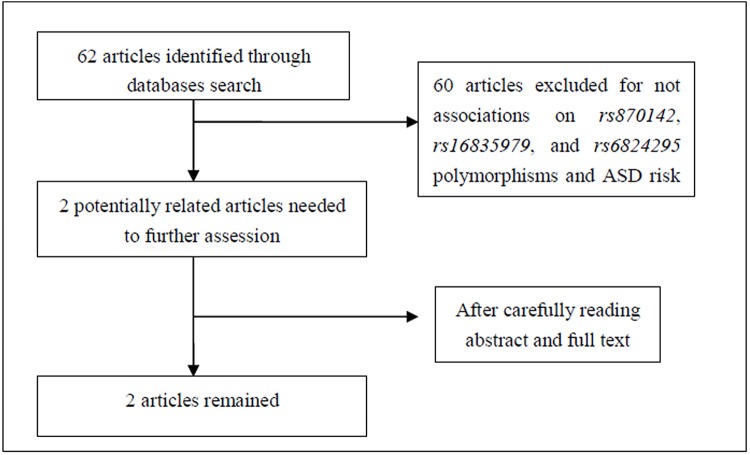
Specific screening flow diagram of articles in meta-analysis.

**Fig 2:**
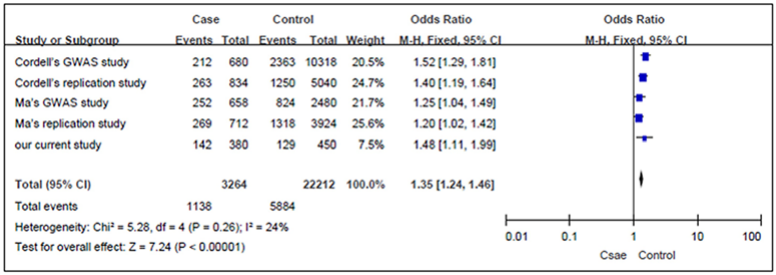
The results of meta-analysis of *rs16835979* polymorphisms and ASD risk.

As shown in Fig 1, a total of 62 articles were searched. After reading abstract and full text, 2 studies met the eligibility criteria, including Cordell’s original GWAS and replication study[9], and Zhao’s GWAS and replication study[13]. The basic information of included studies was shown in Table 4. Zhao’s replication study only reported the association between *rs16835979* and ASD risk in Han population in southeast China. Considering the high linkage disequilibrium among the 3 SNPs, we selected SNP *rs16835979* to conduct a meta-analysis. Our meta-analysis included Cordell’s GWAS and replication study, Zhao’s GWAS and replication study, and our current study, consisting of 1632 ASD patients and 11106 controls. We found there was no heterogeneity among studies, therefore, we utilized fixed-model to assess the associations between *rs16835979* polymorphism and ASD risk in the combined population. The meta-analysis showed that SNP *rs16835979* was associated with ASD risk (combined OR (95%CI) = 1.35(1.24–1.46), *P* <0.00001) in the allele model. Specific results are summarized in Fig 2.

**Table 4:** The basic information of the studies included in our meta-analysis.

Study	First author's name	Year of publication	Study design	Ethnicity of study population	Sample size(case/control)
S1	Cordell(GWAS)	2013	Case-control	European Caucasian	340/5159
S2	Cordell(Replication)	2013	Case-control	European Caucasian	417/2520
S3	Zhao(GWAS)	2014	Case-control	Chinese Han	329/1240
S4	Zhao(Replication)	2014	Case-control	Chinese Han	356/1962

## Discussion

In the current study, significant associations were detected in allele, genotype, and haplotype tests between ASD cases and controls. We found that individuals carrying the T-allele of *rs870142*, the A-allele *rs16835979*, and the T-allele *rs6824295* had a respective 50.1%, 48.5%, and 38.6% increased risk of developing ASD, compared to wild-type allele carriers in our study population. Using a recessive genetic model, the TT-genotype of *rs870142*, the AA-genotype of *rs16835979*, and the TT-genotype of *rs6824295* were associated with increased ASD risk in our cohort. These findings were consistent with the results of the Cordell’s GWAS [9]. Recently, Zhao’s study also reported that the 3 susceptibility SNPs affected ASD risk in a Han population in southeast China [13]. Our study showed that these 3 SNPs were associated with the increased risk of ASD in the Han-Chinese population in southwest China, providing additional proof that these 3 SNPs were related to ASD development. However, probably due to genetic heterogeneity, the minor allele frequencies of the 3 target SNPs in the controls of our study population are higher than those in the European populations, but similar to those in Zhao’s study population [9,13].

In our haplotype analysis, the TAT haplotype was also associated with ASD risk. Given the similarity seen in the ORs and significance levels between the effect of haplotype TAT (OR (95%CI) = 1.54 (1.030–2.380), *P*_*FDR-BH*_ = 0.016) and the individual SNP effects (*rs870142*: OR = 1.501, *P*_*FDR-BH*_ = 0.018; *rs16835979*: OR = 1.485, *P*_*FDR-BH*_ = 0.012; *rs6824295*: OR = 1.386, *P*_*FDR-BH*_ = 0.025, respectively), we conducted conditional haplotype-based testing to determine whether the haplotype effect was attributable to a single SNP effect. Consequently, we found that haplotype association could all be accounted for by any one of the SNPs, probably caused by the close linkage disequilibrium associations among the 3 SNPs. From the significance level, the haplotype association could be most likely caused by the *rs870142*.

Due to our relatively small sample size, we conducted a meta-analysis with the combined data of Cordell’s GWAS and replication study, Zhao’s GWAS and replication study, and our current study[9,13]. Zhao’s replication study only reported the association between *rs16835979* and ASD risk in Han population in southeast China. Considering the high linkage disequilibrium among the 3 SNPs, we selected SNP *rs16835979* to conduct a meta-analysis. Still, we found the SNP *rs16835979* was associated with ASD risk in the combined population.

Although the specific mechanisms by which the 3 SNPs affect the risk of ASD have not been elucidated, all the 3 variants locate in intronic regions of chromosome 4p16, potentially influencing the regulatory function of transcription factors, further interfering in the synthesis of structure proteins. As is described in the European GWAS analysis, SNP *rs870142* locates in the interval between *STX18* and *MSX1*. STX18 is a synaptosome associated protein receptor that functions in the endoplasmic reticulum, intermediate compartment, and cis-Golgi vesicle trafficking and may not be a candidate for ASD risk [14]. *MSX1* encodes a homeobox transcription factor which expresses during atrial septum development, both in mouse and chick [15]. Importantly, MSX1 could functionally interact with TBX5, a transcription factor known to be critical in atrial septal development [16,17]. We suspect that the mutation of *rs870142* may influence the expression of MSX1 and increase the risk of ASD. SNPs *rs6824295* and *rs16835979* locate in STX18 antisense RNA1 (STX18-AS1), a long non-coding RNA. Gene expression studies of STX18-AS1 show that the risk alleles of the 2 SNPs, *rs6824295* and *rs16835979* are associated with lower expression of STX18-AS1 as mentioned in the Cordell’s GWAS [9]. We suspect that the changed STX18-AS1 expression may be associated with ASD occurrence. However, much remains to be done to understand the specific mechanisms of the 3 risk SNPs in relation to occurrence of ASD. Future studies should focus on the association between *rs870142* and the expression of MSX1 and TBX5, to help determine how the *rs870142* mutation affects ASD occurrence; and regarding *rs16835979* and *rs6824295*, on how the changed STX18-AS1 affects ASD risk.

Our study results showed that 3 SNPs at chromosome 4p16 are associated with ASD risk in Han population in southwest China. Our study may be limited by the incapability of performing more genotyping to test effect of the potential population stratification on our study results as well as the small sample size. However, in our recruitment of the study subjects, we only recruited local Sichuan Han people, excluding those who are not local Sichuan people or who are from other ethnic groups. In this way, we did our best to make the case and the control groups genetically comparable. In addition, to avoid the effect of small sample size on our study results, we calculated empirical significance values on the basis of 10,000 permutations for each SNP. Also, we conducted a meta-analysis of published literatures and the results further proved the association between the investigated SNP and the risk of ASD.

Our study and meta-analysis shows that the 3 susceptibility SNPs, *rs870142*, *rs16835979* and *rs6824295*, at chromosome 4p16 are associated with the risk of ASD. The mechanism by which the genetic changes influence the development of atrial septum warrant further study.

## Supporting Information

S1 Checklist PRISMA Checklist. (DOC)
